# A new life with a new language: Russophone immigrants’ reflections about language learning

**DOI:** 10.3389/fsoc.2024.1443762

**Published:** 2024-10-30

**Authors:** Ekaterina Protassova, Maria Yelenevskaya

**Affiliations:** ^1^Department of Languages, Faculty of Arts, University of Helsinki, Helsinki, Finland; ^2^Department of Humanities and Arts, Technion - Israel Institute of Technology, Haifa, Israel

**Keywords:** second language learning, Russophone diaspora, shared opinions, linguistic biography, life trajectory, attitudes toward language learning, naïve linguistic views

## Abstract

**Introduction:**

This research investigates resilience and lived experiences of transnational Russophone families amidst global changes, with a focus on the intricate dynamics of communities spread across borders. The study emphasizes the importance of considering individual migrant experiences in understanding language learning and integration. We explored perceptions of local language proficiency among Russophones; challenges faced by adult Russophones in learning a new language; attitudes and experiences of adults regarding language learning; and strategies Russophone immigrants use to address gaps in the target language.

**Methods:**

Methodologically, the research employs ethnographic and thematic analyses, drawing on a diverse array of sources including interviews, social media posts, and personal communications. This approach highlights the necessity of considering both prompted responses and spontaneous discussions to capture authentic opinions on language learning from various perspectives.

**Results:**

The study underscores the interconnectedness and interdependence within transnational families, illustrating how their lives are shaped by factors that transcend national boundaries. The examination of their diverse experiences reveals their capacity to endure and overcome challenges of integrating into new societies. Russophone migrants’ attitudes toward language learning highlight how learning the host country’s language enhances integration and social mobility, while maintaining the native language preserves cultural heritage, although second-generation immigrants often feel disconnected from their linguistic roots.

**Discussion:**

Studies by various authors discuss challenges which immigrants face when adapting to a new linguistic environment. This project emphasizes the impact of language learning on identity and reveals cultural flexibility in attaining social justice in multicultural contexts. These insights suggest that language programs and policies should address both the practical needs of immigrants and the preservation of their cultural identities taking into account their naïve views about language learning.

## 1 Introduction

In the 1990s, we witnessed mass immigration to Finland, Germany, and Israel from the USSR and the Post-Soviet Space (PSS). These migrants belonged to the category known as *repatriates.* Some were motivated by ethnic pride and a desire to live in their ancestral homeland, while others fled political upheaval, ethnic conflicts, and economic decline (see Fialkova and Yelenevskaya, 2007; Meng, 2001; Protassova, 2004). Due to the Cold War, few had visited their ancestral lands before migrating and had little idea of what to expect. Except for the elderly, the majority of the migrants had Russian as L1 and received education in Russian. Ingrian Finns residing in small villages in Karelia and Leningrad region preserved traces of the Finnish language, while Russian Germans maintained some fractured remnants of dialects. As time passed by, these were drifting further away from standard varieties of Finnish and German. Soviet Jews were among the most urbanized ethnic groups, primarily assimilated and Russian monolinguals. The elderly who grew up in Ukraine, Belorussia and Baltic Republics knew some Yiddish. Mountain Jews who came from the Caucasus could speak the Tat language, and Buchara Jews from Uzbekistan knew Judeo-Tadjik, but almost nobody could communicate in Hebrew, the state language of Israel.

Post-war generations had few opportunities to learn Finnish unless they lived in the Karelian Autonomous Republic or studied it at the few universities offering courses of Finnish. The fluctuating language policies in Karelia during the 1930s and 1940s, documented by Kilin (1999) and Zinkevich (2018), alternated between promoting and suppressing Finnish. Hebrew was proclaimed to be the language of the clerics and Zionists in 1926, so all the cultural initiatives involving the use of Hebrew were devoid of state support (Polian, 2018). Until the mid-1980s, private teaching of Hebrew could lead to prison sentences, which discouraged Jews from learning it. German was one of the popular foreign languages in Soviet schools, but except for specialized schools which used excellent textbooks and offered more than twice the number of class hours as compared with the rest of the schools, the results were very poor. In the absence of opportunities to travel abroad and in the atmosphere of anti-Western propaganda, the mainstream Soviet school used textbooks aiming to transfer Soviet values and the ideology instead of introducing schoolchildren to the cultures of other countries. This acted as a demotivator and “I studied but I have not learned,” was a common situation after five or 6 years of foreign language learning.

Those Russian Germans and Ingrian Finns who hoped that their knowledge of the language of their historical Fatherland would be sufficient at least for the first period of life were disappointed. Their grammar proficiency was either lost over generations of living away from ancestral homelands, or never properly acquired in the Soviet school. Their oral skills were strongly influenced by the Russian language and languages of the Soviet environment. One participant in our project ironically called the language spoken by his peers “Swabian-Crimean-Kazakh-German language.” Written skills of these people were very weak, and they could not differentiate between formal and informal ways of expression.

Until the beginning of mass emigration in the 1990s, adult language learning among citizens of the USSR was not widespread. Foreign travel was limited to the selected few deemed completely loyal to the system. Fiction in foreign languages published in the Soviet Union was mostly classics, and the number of copies put in circulation was pitifully small. Foreign movies were dubbed, and special efforts were made to create noise and interference preventing people from listening to the programs of foreign radio channels whether in Russian or in other languages. Correspondence with foreigners could ruin one’s career, and letters sent from abroad often ended up in the drawers of censors, never reaching the addressees. In fact, there were almost no contexts in which command of a foreign language could have a pragmatic value. The situation changed in the 1990s. Prospective migrants, students, researchers, and tourists all felt that basic English was essential for functioning abroad. Moreover, ideas about the benefits of language learning for developing and preserving cognitive abilities also became a factor encouraging Russophones to start or resume learning new languages when they were past their student years (*cf.* Bulajeva and Hogan-Brun, 2010; Kazakbaeva, 2023).

The Russophone diaspora has been growing over the past 35 years, with generations changing and waves of new immigrants arriving. In different countries there were special programs for accepting qualified labor or inviting migrants willing to be trained for in-demand occupations. The latest influx was associated with Russia’s large-scale intervention in Ukraine. Borusiak (2023) investigated challenges faced by highly educated emigrants from Russia across 26 countries, revealing a gradual communication decline with those who remained in Russia and increasing adaptation to the new environment.

In the last two decades, interactions of the Russophones living outside Russia with Russia’s inhabitants and with Russian speakers residing in different countries have been greatly facilitated by access to mobile devices and to the possibility to keep in touch through social networks (Bassin and Suslov, 2016; Solovova and Vakser, 2023; Vorobeva et al., 2022; Yelenevskaya and Protassova, 2023b). Through the posts in social media, Sippola et al. (2022) studied the significance of embodied cultural capital in enhancing one’s social position in the host country (Estonian and Russian speakers in Finland), particularly through understanding and adhering to its normative rules. They used ethnographic analysis and found that education, taste in clothing, food, and style, and language proficiency emerged as key resources for positioning and making distinctions, while discussions also provided opportunities for participants to showcase their cultural resources. Grebenyuk and Subbotin (2021) examine migrant integration in destination countries, covering assimilation challenges, identity preservation, migration patterns caused by various factors, including natural disasters and political reasons, as well as intellectual and labor migration. They emphasize the uniqueness of data generated by social networks, which provide real-time coverage of the entire population. The limitations of this method are accessibility, representativeness, and user preference variability.

## 2 Aims, material and method

This study aims to determine how language learning affects Russophones’ integration into host societies. While many studies have examined how language proficiency impacts job market success (see, e.g., Chiswick, 2008; Isphording, 2015; Roll, 2003; Zorlo and Hartog, 2018), adult immigrants’ further education, and socio-economic mobility, fewer have explored immigrants’ perceptions of their language skills and attitudes toward multilingualism. This paper aims to fill that gap. We want to demonstrate challenges of the new life with a new language in the naïve representation of the new speakers (De Costa et al., 2016; Hoffman, 1998).

Undertaking this project, we posed the following research questions:

- How do Russophones perceive the importance of local language/s proficiency?
- What challenges do adult Russophones face in learning a new language?
- What attitudes and experiences do adults share regarding language learning?
- What strategies do Russophone immigrants employ to cope with gaps in the target language?

Material for analysis was drawn from various sources, including individual and group interviews and discussions, for which participants provided informed consent to be cited anonymously. Before conducting interviews, all participants were provided with detailed information about the study and its objectives, the use of the data collected, and their rights. Written consent was obtained from each participant, ensuring they understood their participation was voluntary and that they could withdraw at any time without any negative consequences. All personal data provided by participants were handled confidentially. Participants were assured that their identities would remain private, and that the information shared during interviews would be stored securely, accessible only to authorized research personnel. To ensure anonymity, all participants were assigned pseudonyms, and any identifying information, including locations, specific job titles, or personal details, was removed or generalized in the analysis.

We also collected material from social media, using open publications accessible through blog aggregators. For publicly available Facebook discussions, we adhered to ethical standards concerning the use of publicly accessible data. As the discussions were already public, explicit consent from the users was not required. However, we strictly followed guidelines regarding the ethical use of social media data, ensuring no identifiable information was used in the analysis (Townsend and Wallace, 2016; Zimmer, 2010). Although Facebook posts were publicly available, any identifying details such as names, usernames, and other personal information were omitted or altered to protect individuals’ privacy. User identities were anonymized by removing or altering any identifiable features such as usernames, profile pictures, and specific references to locations or events that could reveal the identity of the post authors.

The first three sections of the text are dedicated to Germany, Finland, and Israel because we have studied them for a period of more than 30 years and have accumulated longitudinal data which can be reassessed. These countries embraced ethnically privileged migration, also known as repatriation, and in the first sections, we focus only on these migrants. We translated the excerpts from Russian without editing them and trying to preserve individual style of the speakers.

It is important for us to consider not only what people say on a particular topic when prompted or asked a focused question but also when they initiate the discussion themselves. We believe that in such situations, even if participants are deliberately provoked, sincere opinions on the issues that interest or bother them are likely to come up. We also found it useful to compare opinions expressed on different platforms and in different formats. Naturally, not every lay person is an attentive observer with good analytical skills and able to reflect on language learning processes. Sometimes participants are too shy to give details if they consider themselves to be failures in language learning. Some others add colorful details in order to ameliorate their experiences or reproduce other people’s opinions about language learning. We can assume that most utterances are influenced by memories of how they themselves learned new languages, what worked for them and what did not.

In our research, we primarily used ethnographic and thematic analyses. The latter allows us to systematically identify and organize data collected, singling out topics which emerge in conversations, interviews, essays, and any other data sources the researchers have. This helps us make sense of shared experiences and meanings. In other words, it is a way of identifying what is common to the way a topic is talked or written about (Braun and Clarke, 2012). The ethnographic analysis presupposes both descriptions and interpretation of cultural behavior and practices of the individuals and groups studied. The ethnographic method favors triangulation of data sources and methods. It also uses thick descriptions which provide the context of the experiences analyzed, seek to determine intentions and meanings organizing participants’ experiences (Denzin, 1994; Gubrium and Holstein, 1997; Lazarton, 2003; O’Reilly, 2011). These methods presuppose meticulous work with data sources throughout the project. Writing fieldwork notes and ethnographic diaries documenting communication with the participants or obtaining additional material which represents the phenomena and people in a new light may change the researchers’ initial perspective and ultimately, the final report (Clandinin and Connelly, 1994). Our interpretation of the participants’ words is neither objective nor value free. It is the result of our own subjectivities based on our experience as immigrants, language teachers and researchers of the linguistic and cultural aspects of migration.

## 3 Theoretical background

Adult immigrant language learners are a numerous and growing group, but this category of learners remains under-researched even in immigrant-receiving countries, although interest in the subject is gradually increasing, (see e. g., Extramiana, 2012; Mouti et al., 2021; Krumm and Plutzar, 2008; Mathews-Aydinli, 2008, etc.). Some migrants start learning the local language/s shortly after arrival, but others keep delaying it until their other resettlement problems are resolved. They are unaware that the longer they postpone language learning, the harder it is to integrate into the host society. Immigrant-receiving countries have various approaches to the task of teaching immigrants and refugees (Burgess and Rowsell, 2020; García, 2017). If teaching focuses on skill development, and forms of assessment are rigid and do not consider the learners’ cultural background and prior experience, the students become stressed and intimidated and may lose motivation to continue their studies. On the other hand, if teaching materials are multimodal and model situations that newcomers face in their everyday life, the students’ motivation receives a boost. If a curriculum encourages learners to compare their own language and culture with those of the host country, they are likely to discover striking similarities but also differences. Moreover, at some point, the learners come to realize changes in their own worldview, testifying to emerging hybrid identities (Alim and Paris, 2017). Those migrants who resettle in non-English speaking countries often must combine acquiring the local language/s with learning or improving their English which gives access to the cultural capital across the globe and has become a prerequisite for academic studies and white-collar jobs in many countries (Burns and Roberts, 2010; Strömmer, 2017).

More than with other categories of learners it is essential that adult immigrants and refugees develop autonomy in setting goals, choosing strategies, and taking responsibility for their own studies. Not everyone is ready for this. In some cultures, a teacher-centered approach still prevails. Older people in particular are used to relying on the teacher more than on themselves. Today, thanks to the spread of digital technologies there is an abundance of online materials for self-directed studies uploaded to the net. However, not everyone is able to choose appropriate materials on his/her own, and this is where teacher guidance, monitoring and suggesting alternative paths is important (Grover et al., 2014). Most of the adults who are not complete novices in learning have their own learning styles and have developed some strategies, which they use whether consciously or not. These strategies can be divided into memory, cognitive and metacognitive, compensational, affective and social (Ehrman and Oxford, 1990). Some studies suggest that more proficient learners apply a wider variety of strategies in a diversity of situations (see, e.g., Ehrman et al., 2003). Moreover, a learner equipped with well-tested strategies is better adjusted to self-directed learning than the one who is not.

Besides institutional language learning, it is necessary to mention the role of “incidental” learning that occurs when newcomers start participating in various activities together with members of the host society, be it sports teams, hobby groups, charity organizations or local housing or parents’ committees. Some immigrants see their activities as purely recreational and are not even aware that learning is taking place. Although it is difficult to measure how much additional exposure to the target language and practice in informal contexts contributes to the proficiency, but it does boost the newcomers’ self-confidence and gives them additional knowledge about the culture of the host country (see Alenius, 2017; Rogers, 2017). Moreover, these activities may contribute to more positive attitudes to multilingualism.

An attitude is commonly defined as a psychological evaluation that reflects a person’s positive, negative, or neutral feelings toward an object, person, idea, or situation (Vogel and Wanke, 2016). Language learners’ attitudes play a crucial role in their success and motivation to learn a new language (Alhamami, 2022). Positive attitudes, such as enthusiasm and interest, lead to greater motivation and effort in learning. Negative attitudes like frustration, anxiety, and a lack of confidence can hinder the learning process (Hosseini and Pourmandnia, 2013). Attitudes can predict behavior, but their accuracy depends on the alignment between the attitude and behavior measures (Haddock et al., 2020). Learners’ attitudes are shaped by various factors including their cultural background, previous experiences, the type of language tasks set, and the learning environment. We will analyze both explicit attitudes while investigating interviews, and implicit psychological tendencies presented in blogs and online discussions.

Immigrants’ attitudes toward learning the language of their environment can vary significantly depending on the background, education, prior experience in language learning, motivation, cultural beliefs, and the societal context in which they find themselves. Linguistic needs of the parents differ from those of the children, and in many cases parents perceive their children’s attitudes toward second-language learning as more positive than the children themselves (Mirici et al., 2013). The impact of educational intervention in adolescent immigrants’ practices revealed that integrating different activities into language teaching promotes a positive attitude toward oral practice and greater engagement in the learning process. Ultimately, this facilitates attainment of more satisfactory oral language proficiency (Garrido and Ruiz, 2013). Among adults, variables such as education level, age at migration, and a degree of language exposure through social networks significantly influence satisfaction with the results of the language-learning programs, while a socioeconomic status and gender do not (Reichenberg and Berhanu, 2019). Feelings of moral discomfort or well-being at school predict attitudes toward learning the target language, with academic grit partially mediating this relationship (Altıntaş and Kutluca Canbulat, 2024; Schachner et al., 2017). Troesch et al. (2021) aimed to explore how parental attitudes towards acculturation relate to immigrant children’s second language (L2) skills, identifying factors such as parental L2 proficiency and early childcare attendance as significant predictors. Results demonstrated a negative correlation between parental acculturation attitudes and children’s L2 skills, highlighting the importance of early childcare and parental proficiency in fostering L2 learning among immigrant children. As Janta et al. (2012) put it, the conceptualization of language learning among migrants, as outlined in the adjustment literature, posits a continuum from passive observation to active language use, with stress and anxiety being inherent factors in this process. The newcomers’ capacity to endure stress influences their adoption of an active stance in language learning, while interactions and networks play a crucial role in co-creating knowledge, including linguistic competence.

Transnational Russophone communities of practice encompass diverse networks and identities formed by Russian speakers worldwide (e.g., Byford et al., 2019; Protassova and Yelenevskaya, 2024). These communities are shaped by migration, cultural exchange, and the evolving dynamics of language use in various sociopolitical contexts. Mobility and connectivity play a crucial role in the formation of these transnational communities, where discussions on language use are vital for both integration into new societies and the maintenance of transborder ties.

In recent decades, social networks have emerged as major communication platforms, particularly during the COVID pandemic, which drastically reduced face-to-face interactions worldwide. The growing mobility of populations has further fueled their popularity. Social networks not only help users forge new friendships but also maintain existing ones. The flexibility of both synchronous and asynchronous communication makes these platforms attractive. They are used for sharing opinions, discussing events, lobbying, disseminating information, advertising, and offering support, among many other functions. Online debates can evoke genuine emotions. They assist in problem-solving, and provide crucial information (Tharapos and O’Connell, 2020). In the global Russian-speaking diaspora, social media serves as a vital barometer of public opinion, emphasizing the need for a broad perspective when analyzing these platforms (Ryazanova-Clarke, 2014, 2017).

With the widespread use of platforms like Facebook understanding their usage patterns and implications is crucial, as many people’s offline connections also exist online (Hollenbaugh and Ferris, 2014). Social networks and actual life complement each other, rather than opposing each other (Guizzardi, 2013: 181). Scholars have increasingly used data from Twitter (now X), email, and Google searches to explore demographic trends (Schneider and Harknett, 2022). Platforms like Facebook are becoming valuable tools for research, especially in recruitment and retention, despite concerns about privacy and consent. As social media becomes a standard mode of communication, researchers should consider its potential (Bennetts et al., 2021). Bloggers and participants in discussions on social media behave differently on Twitter, Facebook and LinkedIn (Ermakova et al., 2021).

Social networking is now integral to daily and professional life, offering vast opportunities to connecting with diverse communities. Mansour (2020) highlights how despite diverse parenting practices, a multicultural mothers’ group on Facebook shares information and develops common norms to function effectively. Pötzschke and Braun (2017) found social media recruitment to be cost-effective for targeting migrant populations. Social networks like Facebook are essential for migrants, helping them establish new connections and maintain old ones, manage relationships, and stay informed about social activities. Discussion groups for Russophone immigrants are particularly active, with members sharing experiences on navigating bureaucracy, job hunting, and language learning. While there is research on internet use among migrants, the specific impact of social networking on social adjustment remains underexplored, highlighting the need for further research. However, internet researchers must be cautious of potential errors and ethical concerns (see Reips, 2002).

The use of social networks among Russophone migrants plays a crucial role in mutual support for learning a second language. These platforms provide spaces where migrants can share resources, exchange experiences, and offer encouragement to one another. The discussions in online forums and groups help to fill knowledge lacunae, reduce the isolation often felt by newcomers, and create a sense of community. By leveraging these digital connections, Russophone migrants can access practical advice on language learning, find emotional support, and build networks that facilitate their integration into new societies. This collective approach to learning underscores the importance of community-driven initiatives in overcoming the challenges of acquiring a second language in a foreign environment (Yelenevskaya and Protassova, 2023a).

## 4 Results

In the early 1990s, Finland, Germany and Israel were not equally well prepared for absorbing large waves of newcomers in language courses and often had to employ teachers who had no special training in methodologies of intensive teaching and accelerated learning. Moreover, while the sentence “Language is what matters most” was reiterated by many new arrivals like a mantra, not everyone was prepared to invest much time and effort in language learning. Having mastered the basics and establishing contacts with local residents, many found out that the language spoken in real life differed from the literary norm taught in class and that several months of studies are insufficient to attain proficiency needed to use the language in a variety of contexts. Although language courses for immigrants played their role as a tool for socialization in the host society, some migrants felt they could not afford to study while they were still jobless; others were ashamed to attend language courses because, as they reported, they were adults and “only children go to school,” or because they were men, and only women can sit in class repeating after the teacher. Therefore, many newcomers did not use the opportunity offered by the states to ethnic migrants and sought to integrate into the job market, hoping that they would learn the language “on the job,” interacting with colleagues. What they did not realize was that white-collar and some blue-collar jobs required language skills much more sophisticated than what could be learned in casual everyday interactions. Only minimal language knowledge was required for unskilled jobs. In these workplaces Russophone workers encountered both immigrants from other countries and locals who spoke various dialects and sociolects, exposing them to the linguistic diversity of the target language. Yet, communicating with their colleagues did little for improving their proficiency, thus not increasing their chances for upward socio-economic mobility. Taking care of the elderly provided more opportunities for closer communication with the native population of higher social status who spoke the language of educated classes. Elderly people were often eager to communicate and help their caregivers with language learning. Friendly relationships with neighbors also facilitated integration. A good way to practice and improve the local language/s was taking children to playgrounds, joining handicraft associations, choirs, and various sports clubs, and even dog walking. One participant recounted that in the morning she would listen to a cooking show, then go shopping to buy the necessary ingredients, and then she would watch a replay of the show cooking simultaneously with the host, so she learned a lot with the help of television. Watching programs with subtitles helped to learn not only the pronunciation of what was written but also the spelling of what was heard. Interview participants pointed out that reading a book aloud simultaneously with listening to an audiobook, copying and translating texts helped them.

### 4.1 “Most important is the language”: Russian Germans’ 30 years of experience in Germany

Research on the linguistic integration of Russian Germans aims to examine how this particular ethnic group adapts linguistically to their new environment, typically in Germany. We investigate various aspects such as language proficiency, language use patterns, attitudes towards language learning, heritage language maintenance, and participation in the dominant language-speaking community. This research involves studying factors influencing linguistic integration, such as socio-cultural background, educational opportunities, and language policies. It provides insights into the challenges and strategies employed by Russian Germans in navigating language learning, identity preservation, and integration into their host society (Meng and Protassova, 2016, 2022). About 70 participants were interviewed in their homes, mostly in 2016. The interviews were held in Russian with occasional German insertions in the speech of the interviewees. Here are some excerpts (initials of the participants appear in bold):

**AK**: At home, I speak Russian, at work, it’s German.

**Interviewer**: No problems with German?

**AK**: Well, there are. Still, there are problems. No matter how much you study, you still don’t know everything. [Official] letters come from different places. Difficult words. The words you use frequently, of course, you know them, but those you haven’t heard or don’t know, of course, you take a dictionary or ask the kids what they mean.

**Interviewer**: Do the kids have problems with German?

**AK**: Rarely. As far as I can see, they have no problems:, they communicate, they talk, and they read and write. So, if you read something in German, you know it yourself, but it’s difficult to explain it in Russian, so it’s harder to say it later. But I understand it myself.

**AK**’s assessment of his language proficiency presents a realistic view of the challenges he faces with the German language. He acknowledges that despite studying, there are still difficulties, particularly with understanding complex or unfamiliar words, as well as expressing himself in German. His observation about his children’s proficiency in German indicates a positive outcome of their exposure to the language from a young age. Overall, **AK** provides a balanced reflection on his language skills, highlighting both strengths and areas needing improvement.

Our interviewee **IM:** says that those who did not study in Germany have difficulties finding a.

proper job.:

**IM**: For me, there was a very big language barrier. Because I’m Russian, you know. I came here: no mom, no dad; no uncle, no aunt.

His wife could study; at least she heard some German from her grandparents. At school, **IM** started to learn something:

**IM**: Well, doch [German: however], we had some German there, we learned the alphabet. Yes, I could read and write but with mistakes. There wasn’t such an intensive course there. Besides, I graduated from a rural secondary school there.

His wife joins in to explain:

**GM**: There, in Russia, there were no such intensive language studies as here, in Germany. If you learn a language here, you can speak it, but not there.

**IM**: In the past, I didn’t understand how people could understand German but couldn’t say anything. That’s how I. I understand literally everything in German, but I can’t express it trotzdem richtig [German: nevertheless correctly], correctly. Because I didn’t learn it in school, for me these prefixes, phrases, everything is at the level of everyday language, so to speak.

**GM:** He can’t write all of this.

A bit surprised by himself **IM** observes that his inner monologues have become bilingual and that he speaks to himself in German when he is at work. He reflects on the challenges faced in Germany by immigrants who had no prior language education. He mentions a significant language barrier many newcomers experience, which hinders their employment opportunities. Despite some self-learning efforts, he struggles with expressing himself effectively, particularly in a written form. He feels he needs additional language support to communicate effectively in public settings. His story underscores the importance of language education and resources for immigrants to facilitate successful integration into society.

The **S** family tell us that they often speak German at home and they claim that hey even dream in German. They believe that all their grandchildren will know the Russian language, but they realize that one needs to really want it. If you tell the children, “Please speak Russian,” they will speak it. They realize that knowledge of two languages is not superfluous,

**TS**: Let it be Kazakh, Uzbek, Georgian, Chinese – it doesn’t matter, the more a person knows, the better. Children learn it, and you can’t make it easier to learn. And children, I think, know this; they just don’t want to show it when they go to school, like, ‘You’re Russian, you’re Russian.’ But then, when they grow up a bit, they’ll be a little smarter and will think, ‘Oh, it’s actually good that I can speak Russian.’ When they’re children, they hide it more.

**NS** recollects: At first, it wasn’t easy. After five years here, we were still trying hard to stand on our own feet. At first, I told my mom, ‘I’ll buy a Mercedes here, go back, sell it, and buy my house back.’ Those were my thoughts. At first, I couldn’t come to terms with it because if you don’t know the German language, naturally, it’s hard for you. Literally, after about a year and a half, all of that [hard times] ended.”

**AS:** Still today, I can’t fully understand German literature. I just can’t grasp it completely, so, it becomes uninteresting to me. It’s clearer when you read a newspaper because you know the current topics and what news is being discussed. But in novels there are a lot of words that are unfamiliar, that don’t come up in our everyday life. My husband reads and says, ‘It turns out there are so many beautiful words, but they only appear in novels.’ Yes, unfortunately, we don’t use everything [there is in the language] in our everyday speech. Much remains incomprehensible for us.

The **S** family emphasize that in their household German is used but they consider maintaining Russian proficiency important as multilingualism is beneficial. They stress the significance of encouraging children to speak both languages and appreciating the advantages of multilingualism for personal development. In addition, they admit that children are reluctant to speak Russian in public but anticipate greater appreciation of multilingualism as they mature.

**AS**: At the time, when we arrived here, we didn’t know German, and we wanted our children to learn it quickly. It’s not that we restricted our own contacts with Russian culture. No, we tried not to impose it on them anymore, but rather pushed them more into the German sphere, so that they would learn faster, and we would learn faster together with them. Then, when we already had a bit of a basis, we thought: Russian is also wonderful, and you can’t forget it so quickly because the experience was that older children gradually began to forget the Russian language, the language, that is, maybe not the culture, because children started to communicate more in German. And when these little ones were born, it somehow happened that the daughter was more interested in Russian, not that it was my goal, no, it just happened that she was very eager to take it all in, everything; Russian was interesting for her, and I was happy to give her more of it.

**AS** reflects on her family’s approach to language learning, emphasizing their initial focus on German to facilitate integration, but later recognizing the importance of maintaining Russian culture and language. She describes a shift towards embracing Russian more openly, influenced by her daughter’s keen interest in the language and culture, illustrating a balanced approach to multilingual upbringing.

**LR**: Fear. Yes, I, for example, was afraid. We arrived almost with zero knowledge [of German], and it was especially hard for **OR**, when she went straight to school, the next day after our arrival. She would come home in tears, saying, ‘Mom, I feel like a sheep, let’s go back home, I feel like a sheep here.’ And when my husband said, ‘I don’t understand anything here, I want to go back home,’ it created a terrible atmosphere for us. All the time I wished we could forget the Russian language. We shut our mouths, and we spoke only German. It was fear. Then our child would come [to talk to us], and for about a month she would say, ‘I don’t want to [be here], why did we come here? I don’t understand anything, let’s go back home.’ She wouldn’t say we were home, she always said, ‘Let’s go back home.’ And this fear … so, we forced them, ‘No, don’t speak Russian, come on, say it in German now,’ and they would speak broken German, and when we realized they were already speaking, we would say in German, ‘If you speak Russian the whole day today, I’ll give you a mark. And they started saying, ‘Mom, no, we don’t want your mark. We can’t.’ When younger children were born, the fear was gone, we were already on our feet. So, we failed to notice that they’d forgotten their Russian.

**LR** candidly recounts the fear and challenges her family faced upon arriving in Germany with little knowledge of the language, resulting in a difficult atmosphere and reluctance to hold to Russian culture which they felt to be their own. Despite initially enforcing German-only communication out of fear that they would not be able to settle properly, she acknowledges the negative impact it had on her children’s emotional well-being and language retention. Ultimately, she recognizes the need for a balanced approach to language learning and cultural integration, highlighting the importance of addressing fears and fostering a supportive environment for linguistic development.

As we see, the Russian-German families emphasize the importance of multilingualism, encouraging their children to speak both German and Russian. They reflect on the challenges of language learning upon their arrival in Germany, initially prioritizing German but later realizing how much they value their Russian heritage. They recount the fear of failure and difficulties their families faced due to a language barrier preventing them from feeling relaxed in Germany. They underscore tension and the emotional toll they had to pay to achieve German proficiency at the expense of Russian. When the period of anxiety was over, they came to realize the importance of being proficient in many languages and cultures.

The current political situation differentiates the Russophone speakers in a new way (Hansen and Olsen, 2020; Ryzhova, 2024; Sablina, 2023). One of the Russophone Facebook users said: “Russian Germans DID NOT TEACH their children Russian; they believed they had become Germans, as the German government promised they would be. They gained [German] citizenship immediately, which also played a role in this. And then they discovered that no one considered them German—neither their language nor their mentality supported this. And they found themselves NOWHERE. This is also one of the primary causes for their ‘passion for Russia.’ Other ethnic groups are experiencing similar problems.” Indeed, only those who are grandparents today probably think like this, but their children who have already become parents are not like that (see Protassova and Reznik, 2023).

### 4.2 Evidence from the group interviews in Finland

In the early 1990s, teaching Finnish as a foreign language was a rare specialty, and the concept of Finnish as an L2, i.e., learning it as a language of the environment, was not yet known in Russia. Today, there is a vast number of textbooks available, and learners actively exchange opinions on where, how, with whom, and using which materials it is best to study. Immigrants attend free adaptation courses or enroll in paid ones, preparing regularly over several years or intensively and purposefully for exams. Their learning strategies are widely studied (e.g., Seppälä, 2022; Tammelin-Laine and Martin, 2015). Applying for citizenship one has to take a test. The issue of proficiency in the Finnish language and immersion in Finnish life is considered a crucial aspect because the alternative often leads to susceptibility to Russian mass media, which can be dangerous for societal attitudes (Davydova-Minguet et al., 2019; Khalimzoda and Siitonen, 2022; Protassova, 2022).

In 2023, we conducted interviews with 23 Russophone immigrants working in the Finnish education system. The interviews were held at the participants’ work places. All of them had become competent and proficient enough to receive education in order to work in different capacities in the education system and found their place on the job market. Still, when they have to deal with various institutions and bureaucratic procedures, they rely on their children or acquaintances to translate for them or proofread their written texts. Here are some of their thoughts.

Some began their occupational life in Finland by doing unqualified jobs where they could learn some language. Those who initially worked as cleaners considered it as a steppingstone, as it was not the limit of their aspirations. They continued their education, choosing a profession that would be in demand in Finland, ensuring they would not remain unemployed. One of the female participants could not learn Finnish and even obtained a certificate stating her inability to do so.

An Estonian-Russian teacher, **HJ,** who worked in the Finnish environment, felt satisfied that she could help a multilingual child and understand the needs of someone who feels that she is unable to comprehend everything:

**HL**: Once there was an Estonian girl who spoke Estonian, and it was a great support for her that I knew the Estonian language. Her mother even remarked on how good it was, as she didn’t expect to find that here, and for me, it was heartening that I could practice Estonian because over time you forget; when you don’t communicate for a long time, you start to forget. If she didn’t understand some instructions, I translated them for her, explained what she needed to do, as her mother requested that she should be spoken to in both languages. You see how the child gets involved, knows everything, and can do everything.

Another female participant told us that observing educational institutions from within she had numerous ideas and suggestions as to how to improve various aspects in the education system Yet, she never dared speak or write about them because she was afraid of making mistakes in the Finnish language. Some migrants gave accounts of challenging experiences posed by the language barrier in the workplace. They prefer the strategy of relying on colleagues for accurate translation to prevent misunderstandings and errors.

**OP**: It is difficult to work because of the language barrier, as I might misunderstand or say something incorrectly, so I turn to my colleagues to translate everything accurately and make sure I avoid mistakes, ensuring that everything is done correctly.

The following passage reflects the **LP**’s experience working in a Finnish kindergarten, highlighting interactions with the children and their role in language learning. She recounts instances when children corrected her language mistakes and emphasizes the importance of hands-on learning experiences. This participant mentions the rewarding aspect of seeing the children’s progress and their ability to handle tasks independently thanks to her work and guidance of her Finnish colleague. This example illustrates the dynamic nature of language learning and the valuable contributions of young learners in the process:

For five years I worked at a Finnish kindergarten. The same children, eating the same way, playing the same way, but my knowledge of Russian was not useful there, as there was only one Russian girl with whom I could read fairy tales, talk about something, go outside, and discuss nature. The children were in a preparatory class; they asked me about trees, and when I didn’t know, they would say, “But you’re a grown-up lady, why don’t you know?” I explained that in my language, I know that it is called береза [birch], but I haven’t learned it yet in Finnish. A boy suggested looking up the name of a bird in a dictionary. I told him I would check what the bird is called, and when I came back, he asked if I’d found out. Then he brought me books about dinosaurs, which he loved. He would say, ‘Read, I’ll correct you.’ I would read, and he would listen, then ask me to read again if he didn’t understand. He taught me many things. There was also a girl who would correct me whenever I confused ‘pulla’ [sweet roll] and ‘sämpylä’ [bun]. When I said we had ‘pulla’ today, and she was three years old, she would say, ‘No, today we had sämpylä.’ If I said sämpylä, she objected, “No, today is pulla.” She exclaimed, “How many times do I have to tell you, the sweet one is ‘pulla’, and plain bread is ‘sämpylä.’ She was always correcting me, and I didn’t understand the difference. These kids are our best teachers. Therefore, you have to go with the kids to learn the language, twist it.

This anecdote is an excellent demonstration of the informal and organic nature of language acquisition in immersive settings of the real-world. It highlights the role of social interactions, the humility and openness required for learning, and the dynamic between adults and children in the learning process. The example shows that even in unplanned or unexpected circumstances, language learning is possible and is enriched through everyday conversations and corrections.

To conclude this section, we can say that there are challenges posed by the language barrier in the workplace, and some rely on their colleagues for accurate translation to prevent misunderstandings and errors. Others underscored the role of children in language learning through hands-on experiences and interactions, as well as the mission of their own children to help with translation of documents, filling out forms and checking letters for grammar and spelling mistakes.

### 4.3 Learning Hebrew in Israel

Teaching Hebrew to new immigrants started even before the creation of the State of Israel in 1948. Since Israel’s Jewish population doubled between 1949 and 1951, the task of teaching Hebrew intensively to facilitate the newcomers’ joining of the labor force was essential for the economy of the young country. Moreover, shifting from the languages of the Diaspora to Hebrew was the cornerstone of Israeli ideology and the main way to try to unite people coming from a variety of cultures. Everyone was expected to stop using native languages even in home communication, even at the expense of losing intergenerational ties. Initially, courses that came to be known as *ulpan* (studio) were meant only for immigrants with academic degrees, but gradually, they became accessible to all the newcomers. Today, the full program includes 495 academic hours and is divided into six levels of proficiency. The curriculum and teaching materials are supervised by the Ministry of Education. State-run *ulpans* are free, but immigrants can choose private courses and for the economically weak they are subsidized. Those who already have jobs can opt for evening *ulpans*, and there is also an option for blended and online learning. At the end of each level there are exams and those who pass them receive certificates. When immigration is on the rise, there are separate groups for medical doctors, engineers and the elderly. Many immigrants do not complete all levels but when they feel they have learned the basics they prefer to enter retraining courses and focus on the learning of professional vocabulary while learning a new trade. Studying at an *ulpan* helps newcomers familiarize themselves with Israeli culture and customs thanks to the choice of teaching materials and extra-curricular activities (Golan and Muchnik, 2011; Haramati, 1966; Raijman et al., 2014).

Ex-Soviets who migrated to Israel in the 1990s were mostly monolinguals, including those who lived in multilingual Central Asian, in the Baltic republics, and in the Caucasus. For most of them learning a language to communicate but not only to translate texts with the help of a dictionary was an entirely new experience. Attitudes of these people to language learning were investigated by many researchers (see, e.g., Fialkova and Yelenevskaya, 2007, 239–266; Niznik, 2003; Remennick, 2003). After some lull in the first decade of the 21^st^ century, immigration to Israel from the PSS continued at an increasing pace after the annexation of the Crimea and even more so after the Russian invasion in Ukraine. So, in this section we present views of the old timers who have lived in Israel for over 30 years and of those who are still relatively new in the country. The discussions took place on Facebook in 2023. Several dozens of participants exchanged their views; in the text of the article we only retained information that was publicly available.

A female participant envies those who seem to pick the language up easily as if it were the most natural thing to do. She complains that Hebrew is particularly challenging for her: it goes in one ear and out the other.

It’s very difficult, it just doesn’t come to me. I cry every day, why not Yiddish, it’s almost like German! My German has long been forgotten, but I still understand Yiddish. And Hebrew – it’s agony. I can’t find any clues or logic. When I was learning Italian, I felt enjoyment. English – it’s all around in the air. But Hebrew.

A frequent metaphor used by migrants talking about language learning is “language that sticks to you.” But according to another female participant, only few individuals endowed with special aptitude for language learning are privileged. For others it is hard work. As an alternative to studies, a female discussant recommends immersion in the environment where the target language is spoken. To make her statement more convincing she confides that she has no particular language abilities, yet she managed to learn as much as she currently needs in order to function in the new environment. She is not giving up and thinks that *n a year or so*, *I’ll need to know it a bit better*, *and I will because I have no choice*.

Immigrants often argue as to whether Hebrew is a “difficult language,” ignoring that perception of difficulty depends on whether the target language is close or far from the learner’s native language. Opinions differ, and some try to convince their virtual interlocutors that Hebrew has simple grammar which is logical, and that is why it is relatively easy for people with a mathematical mindset. One of the participants suggests that learners should familiarize themselves with conjugation types of the verbs which serve as models for constructing words: *By getting acquainted with the root of a word through these models, you can build many new words and even guess their meanings.* This utterance triggers multiple comments expressing opinions and suggestions:

- Unfortunately, I am not a mathematician, I am in humanities, and this is my essence.

- Then try to find a suitable method for yourself. Say, if you enjoy singing, try learning through songs. This works for many people. Build word associations… I tried it for learning German articles.

Advice and recommendations which adult learners give to their peers testify to their awareness of the utility of online resources and to their ingenuity in choosing platforms and methods that are suitable for their learning styles. There are many requests to recommend private tutors and companions for practicing Hebrew in informal situations. This does not mean that new immigrants are only looking for contacts with native Hebrew speakers. One of the users posts in a popular group launched by immigrants but frequented by old-timers too that she is looking for a *study buddy*. Note that she uses the English phrase for the sake of language economy since there is no Russian equivalent for this phrase:

Hi! I’m looking for a study buddy to practice conversational Hebrew together twice a week and regularly. My level is gimel-dalet [the names of Hebrew letters which stand for 3 and 4]. The idea is to talk and correspond only in Hebrew. We’ll discuss videos/podcasts/articles, and new words. It is important that the person should be motivated. I know how well it works and how much it helps in advancing the language.

The invitation was enthusiastically accepted by many participants, and most of them emphasized that they were highly motivated. At the same time, there were others who wondered who would correct their mistakes. The conversation clearly shows that some adults are hindered by fear of errors and prefer the teacher-centered approach to language learning, while others are more interested in developing fluency. Among the latter, many mentioned or implied that they had learned other languages by prioritizing fluency over correctness:

This is a wonderful idea! From my own experience I know that it’s not at all necessary for someone to correct your mistakes all the time. When you are absorbed in conversation these corrections will be forgotten anyway. It is important to be able to express your thoughts and emotions and practice small talk. Mistakes do not disturb at all, and they can be cleaned up later. Good luck!

Some participants in this thread invite others to join online chat groups which they created to practice Hebrew, others who have grown up in Israel offer their help in “start talking.”

A popular theme in the groups we monitored is learning Hebrew for professional purposes. Participants exchange information about availability of courses for health workers and engineers, accountants and jurists: *There is a course “ivrit taasukatit”* [Hebrew for employment] sponsored by *Klita* [informal Russified word for Hebrew “absorption” referring to the Ministry of Immigrant Integration]. *It is without any professional specialization but with lexis and studies of the situations which you encounter when you apply for a job, go to an interview,* etc.

Discussants agree that in the worst case, if no course is tailored to one’s field, it is possible to get hold of authentic documents, read and translate them, and memorize words and phrases.

A special case is IT professionals. Those employed in the industry are convinced that it is not Hebrew that novices should worry about but English: *In IT 80% of the professional lexis is in English, the remaining 20% is everyday Hebrew used to connect terms.* To emphasize this point, an old-timer shares his experience:

Once there were Hebrew classes in the program of computer courses, but when I started working, none of my Hebrew-speaking colleagues could understand what is mekhorer [hole puncher], mikledet [keyboard] or orekh yahasim [length of a relationship].

This example is relevant for any language learner studying to feel comfortable in a workplace. English terminology dominates in technology, and starting job hunting Russophone immigrants sometimes prioritize English over the language of the host country. In one of the threads a new immigrant asks advice as to how to improve her English in Israel without using Hebrew. The question sounds naïve, in the country where there is abundance of Russophone English teachers and native English-speaking tutors who do not use translation at all. Some participants in the thread suggest working on both English and Hebrew simultaneously, yet others discourage such endeavor as a source of confusion.

Another frequent theme is the issue of age for language learning. While for some immigrants advanced age is an excuse that getting older people have few chances of becoming proficient in a new language, others claim the opposite, and in both cases, they often cite the story of their mothers to prove their point:

- My mom has been struggling with Hebrew for over 30 years now, despite attending ulpan and various other classes. She even studied with textbooks and tapes on her own. But it just doesn’t come to her. Interestingly, she mastered all the terminology in mathematics, she is a math teacher, in her first year in Israel. But when it comes to conversational language, it’s a different story…

- My mom came here when she was over 60, and she learned Hebrew, and read more books in Hebrew than I did. Admittedly, she had a knack for languages – she was a translator from English. She would get very upset when people in hospitals tried to speak to her in broken Russian.

Two things are of interest here: it often happens that professionals determined to reintegrate into the job market make every effort to learn relevant terminology and pragma-linguistic aspects of professional discourse but fail to master Hebrew used outside their workplace. Secondly, for the elderly, learning a new language is important to be independent and not to suffer from the inferiority complex and patronizing attitudes of others.

Discussion participants criticize those who are unwilling to make efforts to learn Hebrew. The three possible reasons named are laziness, a wish to re-emigrate and patronizing attitudes to a language which is not global. In fact, Russian speakers, in particular those in the metropolis are often blamed for imperial attitudes to other languages and cultures.

Well, I don’t have any special complaints about those who, in our case, don’t want to learn Hebrew. And it seems to me that it’s not always about imperial snobbery. For many, it’s simply laziness because they hope they can get by without it, living in a bubble. There are many people like this in Israel. They could have learned Hebrew much better and integrate much better, but they’re just too lazy. Some people don’t want to engage in sports or take a civic stance. Well, okay.

Note that unwillingness to learn the language of the host country is viewed as similar to reluctance to actively participate in the social life of the civic society.

One of the factors named as an important reason to achieve Hebrew proficiency is helping children at school. It is well known that immigrant parents often feel lost in the first period after migration and have an impression that their children understand the host country better than them. In some sense there is a reversal of roles, which may be stressful for youngsters and backfire later:

But how can they help their children at school when all official papers are in Hebrew? Later, the kids will grow up and go to psychologists complaining that their parents couldn’t help them, that they themselves needed to help their parents.

Learning new languages people often turn into folk linguists. They analyze how they make use of their linguistic repertoire in different communicative situations, what triggers their progress and regress and what metamorphoses occur with the languages learned earlier:

It’s incredibly interesting what happens in our minds! Who would study and explain these language intricacies! I mix up Hebrew and Russian when I’m extremely tired. I can address Hebrew speakers in Russian and don’t even notice which language I’m speaking. At work, they already know this and laugh hysterically!

As members of the one-and-a-half and second-generation immigrants come of age, the number of mixed marriages is growing, and family communication reminds one of an intricate tapestry, with frequent code-mixing and translanguaging, and different family members choosing different languages to communicate with each other:

I came to Israel in 1990 from Almaty when I was 38. I was convinced that learning Hebrew was the top priority for the whole family. What saved me was allowing myself to “relax” and not worry about speaking “right or wrong” My husband also studied diligently but remained silent for a very long time. It’s well known that the male ego often prevails over the female ego. Today, my Portuguese daughter and grandson practice Hebrew on the bus on their way to school, so that my grandson can communicate with his Israeli cousins. He’s only 10, and his Portuguese is at a native level. He knows Russian and English very well, and his Hebrew is decent. At school he has also started learning French as a second foreign language. During all our visits to our children in Porto – for about ten years now – we came to realize that the Portuguese don’t care much for English. Our Portuguese son-in-law, who is fluent in French which he uses at work, started learning English in order to communicate with me. As a result, he knows English perfectly well, but we can’t communicate because while I understand him, when I start speaking. English in my mind is replaced with Hebrew, and sometimes, quite oddly, Kazakh words pop up. This has happened a lot recently, perhaps ‘due to old age.’ When we arrived in Israel, English emerged in my brain in an incredible volume that surprised me, but after I mastered Hebrew at a decent level, it plunged into inaccessible depths. And when I started writing this comment, I meant to say that today the attitude toward English in Portugal has noticeably changed in a positive way, especially among the youth.

This passage reflects on the complexities of language learning and multilingualism within a multi-generational family. The author emphasizes the importance of learning the local language for integration while acknowledging different approaches between genders. It also highlights the unexpected ways in which multiple languages can influence one’s ability to communicate, especially later in life. The changing attitudes toward English in Portugal, particularly among younger generations, contrasts with the participant’s earlier experiences, illustrating how language preferences can shift over time.

Most of the Russophone Israelis participating in the quoted digital exchanges value multilingualism, and those who have attained Hebrew proficiency are proud of themselves and are willing to share methods which they claim to be effective. Participants are even more proud of their mothers who managed to learn the language despite their advanced age upon arrival in a new country. Note that nobody speaks of fathers being successful language learners. For some continued learning of new languages has become a habit and joy, and in mixed families it is a necessity if family members want to maintain intergenerational ties and communicate with their grandchildren and in-laws.

### 4.4 Recent migration wave: better equipped for learning languages?

As mentioned earlier, for many decades of the Soviet power there was no stimulus for the country’s population to learn languages. Due to the inaccessibility of foreign travel and foreign mass media Soviet people were not motivated to learn languages spoken outside its borders. Aggressive Russification made learning titular languages of the Soviet Republics unattractive for Russophones residing in these republics. Learning new languages as a way of personal development was not encouraged, and even those who would like to engage in it could find courses only if they lived in big industrial cities. The situation changed dramatically with the beginning of mass emigration and lifting of restrictions for foreign travel in the 1990s. Russophones did not only find motivation to learn new languages, but different methods of language learning, exchange of experiences became a popular topic of formal and informal discourse. In this section we introduce the reader to the multiplicity of themes related to language acquisition and frequently discussed by members of the Russophone diaspora. These discussions go beyond language-learning tips, but deal with such important issues as intercultural communication, respect of the *other,* intergenerational ties, the value of multilingualism in the contemporary world and others.

In May 2023, a popular Russophone blogger writing about different life and political events sets the stage by asserting that learning the local language is a sign of respect and an essential part of integration, regardless of how long one intends to stay in a new country. She criticizes those who refuse to learn local languages, such as Russian speakers in the Baltic countries, or earlier immigrants to Israel and the USA, who only learned a minimal vocabulary for daily activities like shopping. The blogger’s position reflects a broader belief that language learning is not just a functional necessity but also a cultural and moral obligation for immigrants. This perspective makes salient the tension between maintaining one’s linguistic identity and adapting to the norms of a new country, a theme that recurs throughout the discussion. The former colonialists did not learn the languages of their colonies, and many tourists do not use languages other than English in France, Italy, or Spain. For Russophones, learning Armenian, Georgian, Kazakh or Uzbek, which are languages of the countries where they live, should be a must, but it is not, because they can still cope there with Russian only. Some people who relocated from Russia after the full-scale invasion of Ukraine wanted local people to speak Russian in their presence although they live their own life in the countries of their own.

Several hundred participants took part in the discussion launched by the blogger referred to above. In the comments, a wide array of perspectives emerges, ranging from pragmatic advice on how to learn languages to deep reflections on the social and cultural implications of language learning. For example, one polyglot participant emphasizes the importance of learning phrases rather than isolated words to foster functional communication. This pragmatic approach to language learning prioritizes real-world usage over theoretical knowledge, illustrating the importance of practical application in language acquisition. This resonates with opinions of many adult learners who strive to achieve basic fluency for everyday tasks rather than mastering the language in its entirety.

Participants discuss how many words one needs to know in order to start speaking. The goal is to communicate effectively and understand people, prioritizing functional language skills over theoretical knowledge. Participants agree that it is vital to grasp the meaning of idiomatic expressions and use them in grammatically correct utterances. While over time proper immersion into the target language medium helps with phrases and idioms, achieving fluency in everyday conversations may require active vocabulary of around 3,000–5,000 words.

Commentators touch on the complexity of phonetics in languages like Portuguese compared to Spanish, and they highlight the challenges of aural comprehension. They discuss the utility of additional Slavic languages in facilitating understanding across related languages and reflect on the unique linguistic characteristics encountered during language learning experiences. One person says that *Czech could be covered by a six-month course with feedback for a million bucks!* Another one adds that *to a native speaker of an East Slavic language (Russian or Ukrainian), Czech is completely opaque; even I can hardly understand it*. The third one remarks: *On our first visit to the Czech Republic in 1999, we were surprised by how much Ukrainian helped us understand Czech*. The next participant summarizes this theme jokingly: *Russian plus Ukrainian – they help to understand most of Belarusian when listening to a bit of Polish and Czech*, highlighting the challenge of mutual intelligibility among Slavic languages.

Participants think that those who do not learn the languages of their environment have a kind of impoverished life and deprive themselves of numerous opportunities. They remark that in Germany, there are plenty of Russian speakers who do not even try to learn the language, as they can always find Russian-speaking doctors, lawyers, and experts in any domain. The participants wonder how one manages to live like that. Another member of the discussion remarks that this happens everywhere with large immigrant communities, for example, with Chinese diaspora in Rome [others mention the Japanese in Düsseldorf].

A different opinion is offered by a male participant:

The most important thing is not even the language itself. The crucial aspect is not to oppose

Yourself to the local people and traditions, not to serve your ego elevating yourself above them, but to accept this country and everything in it. For example, in Israel, don’t eat a pork shish kebab on Shabbat, and in Italy, do not try to order a cappuccino after dinner. As for language, it depends on what you need and what resources you have. If full integration is not your goal, if you can generate income without being tied to the country, and if the language doesn’t come naturally, why force yourself? It’s okay to always remain somewhat of a foreigner, to accept that there are limitations in communication, and just live peacefully.

This opinion reflects a pragmatic approach to language learning and integration, emphasizing.

cultural adaptation over linguistic mastery. The participant suggests that while language is important, it may not be essential for everyone, especially if full integration into the local society is not the goal. Instead, the focus is on respecting local customs and traditions, blending in with the community, and accepting limitations in communication without forcing oneself to achieve fluency. This viewpoint recognizes that individuals have a variety of needs and offers a more flexible perspective on integration, suggesting that peace and contentment can be found without complete linguistic immersion.

The discussion touches on the perceived status of different languages. One participant raises a naïve question: *Do you believe that all languages are equal?* and starts comparing Greek and Lithuanian. Some commentators mention that they enjoyed learning Greek when they moved to Cyprus, others learned it for pleasure. A resident of Cyprus remarks: *I do not want or plan to integrate into the local society. English is almost like a second official language here, so there’s no real need for Greek. I’m integrated into the global community, and it’s more important for me than the local one.* Another participant thinks that it is not about languages, their social equality, or finding pleasure in learning and using the language, but it is about respect or disrespect for the country that accepted you. Other participants retort that the perspective on language learning differs from individual to individual. With only one life and limited time, they are uncertain if they would prioritize learning a small language like Lithuanian solely out of respect, especially if it would mean attrition in Italian and German that are in their repertoire. Moreover, they do not perceive integration into local life as necessary, since it would always be overshadowed by the importance of integrating into the global community, extending from the USA to Singapore. As for Lithuanian, if one could live without learning it, they would not bother. Participants question whether it makes sense to regard Greek as superior to Lithuanian, and criteria for such judgments are explored. Yet someone notes that there are individuals willing to learn languages like Hungarian or Lithuanian while living in Moscow, and not even motivated by work necessity or the prospects of immigration, but just because they like these languages. Lithuanian is praised for its beauty, even likened to Elvish. Reflecting on personal experiences, someone who worked hard to learn Hungarian regrets not to have invested equal amount of time and effort in learning Spanish, German, or Italian instead, highlighting the ongoing debate among philologists about language equality.

One woman confesses:

I feel hesitant to learn other people’s languages. In a sense, I feel it’s disrespectful to struggle through a dozen words, straining my interlocutors to understand me, when there’s English (or Russian) that we both know equally well. But I’ve learned that it’s not the same for everyone. Communicating through facial expressions, gestures, and simply because I really want it to feel normal to me. It’s precisely mangling a foreign language that feels awkward. By the way, there have never been any problems – people always try to communicate in a friendly and cheerful manner, so the main thing is not to look like a know-it-all. But in America, it was awkward, indeed.

Discussants agree that integration into a local society presupposes newcomers’ interest in engaging with the local community; hence, a lack of language learning suggests indifference to becoming part of the receiving society.

Participants exchange their opinions about requirements and challenges of language exams. Some fail to understand why in some mixed families spouses do not learn each other’s language, as a result depriving themselves of rich and versatile verbal communication. Others complain that it is difficult to learn a local language when people around you switch to English whenever they hear your accent or watch your hesitation in choosing suitable words.

In March 2024, a Facebook blogger living in Latin America tells her readers that having opened a grammar reference book to clarify something, she suddenly realized that she was no more nervous about her Spanish. She knows that she speaks poorly and is slow to recall words, but after she got her certificate and her host country recognized her Spanish as sufficient for residence, she can improve it just for her own benefit but not because there is an exam ahead urging her to keep studying. She asked whether other immigrants felt the same ambivalence, and many answered affirmatively. One commentator wrote that she wanted to know the language of her host country on many levels and be comfortable with different registers and genres, be it bureaucratic documents, literary texts, colloquial conversations, the Bible, the country’s Constitution, playful songs, ancient legends, Casanova’s adventures and others. She is not sure she needs this, but she wants it. One participant admits that in her work with children, she feels disarmed. She cannot capture their attention, especially in difficult situations. She cannot speak fluently and confidently and struggling with shyness she speaks softly when she needs to be loud and assertive. Despite encouraging the children to correct her, she makes mistakes and suffers when she realizes this. To add to her frustration, one mischievous child mimics her, and this acts as if he *closes a valve. I’ve also noticed something interesting. To feel good about myself and stop getting nervous, I need some time to “warm up.” The longer I speak, the better.*

Three participants complain that they keep forgetting words of the language of their environment; moreover, they feel that their native Russian is deteriorating. They realize that their overall level of education is low and that this is an additional obstacle: *In general, the more languages I could potentially speak, the closer I feel to being speechless*, says one. *If someone speaks a lot or quickly, I start struggling and stop comprehending*, says another. *I remember my own language difficulties and get scared. But since I constantly keep track of the country’s political life, I write down and memorize new words and phrases for myself*, says the third.

Discussants are convinced that those who are language teachers should know the language perfectly, but for all the others attaining even the basic level of proficiency is an achievement. Those who have lived abroad for more than 20 years are embarrassed if they receive compliments as to how well they speak the language. They suspect that they are praised because they are perceived as new immigrants, rather than veterans who should have already attained a native-like level of proficiency. Children or their spouses correct them and often have to serve as their interpreters. Some complain that they keep repeating the same mistakes. It annoys and upsets them when they catch themselves making errors.

In another discussion posted on Facebook in October 2023, a teacher said:

Indeed, it’s a unique period. Georgian, Armenian, Uzbek, Azerbaijani, Kazakh, Kyrgyz, and Baltic languages are in demand. Russians running away from the war began learning these languages. Who knows, perhaps this marks the beginning of a new era in the field of language learning and intercultural communication. What we lack is openness and understanding of others, especially regarding our closest neighbors.

This statement underlines the specificity of the current moment in which previously overlooked and undervalues languages spoken in the post-Soviet space are gaining significance. It underscores the potential for increased cross-cultural understanding and empathy through language learning.

Challenges faced by adult learners, such as psychological stress, disappointment with slow progress, realization that learning a new language is difficult, especially compared to children’s much faster progress often lead to frustration and feelings of inadequacy. This does not only cause a loss of self-confidence but overshadows attitudes toward language learning and hopes for successful integration. Participants emphasize the importance of community, solidarity and mutual support in overcoming these difficulties, with social networks and community events playing an important role in providing resources, practical advice, and emotional support.

## 5 Discussion and conclusion

This article focused on studying attitudes toward language learning among Russian speakers using a comprehensive research approach that incorporates various qualitative methodologies and techniques. The Russophone immigrant parents and teachers in various discussion forums cover a range of relevant topics, such as perceived importance of language proficiency, motivation for learning, challenges confronting adult learners, and attitudes toward multilingualism. To increase validity of our findings we relied on data coming from a variety of sources. We conducted qualitative interviews with adult Russian speakers to investigate deeper their attitudes and experiences in language learning. We analyzed oral and online materials produced by adult Russian speakers, such as social media posts, forum discussions and personal blogs to identify recurrent themes, opinions, and attitudes related to language learning using ethnographic and thematic analysis approaches. Through online communication concerning the life-hacks how to learn languages of the surroundings, adults improve their metalinguistic awareness and interaction skills, construct naïve linguistic theories and experience psychological relief. By integrating multiple data sources and analytical approaches, we captured both the breadth and depth of language attitudes within the immigrant Russian-speaking communities.

The concept of life in a new language was already discussed by many authors, including Soler and Zabrodskaja (2017) and Piller et al. (2024). They describe both the challenges of rebuilding their lives in a new cultural and linguistic context and their resilience in the face of racism and hardship, with implications for language services, migration policy, and social justice. The studies collected by Simpson and Whiteside (2015) offer valuable insights into responses to linguistic and cultural diversity and the shifting mobilities of the twenty-first century, as migrants move across different scales. These range from national political arenas to local affairs, from national to supranational institutions, and down to local classrooms, teachers, and specific groups of adult learners. Angouri (2014) explores multilingualism in the workplace as a multifaceted phenomenon, examining language policies, tacit practices, cultural norms, and the impact of language in professional settings. She addresses the relationship between macro-level policies and micro-level interactions, as well as the commodification of language in diverse workplace contexts. Notably, our study is limited to subjective opinions on the language learning processes in a new country.

Participants in our study believe that those immigrants who refuse to learn the language/s of the host country show disrespect for its culture, so disdain and hostility which confront them should not surprise them. Some are proud that they can use the newly learned language every day and in multiple situations: work, correspondence, telephone calls and entertainment. They are unanimous in stating that once they feel comfortable with the language, their quality of life improves. At the same time, those who relocated, live in resorts and find themselves in expats’ communities manage with international languages. However, even among them there are enthusiasts who try to learn any language they encounter, even if at a very basic level. Those who feel that the knowledge of the local language/s is not a necessity, still agree that it deepens the understanding of the environment, the country’s way of life, history, and hidden problems. Moreover, it helps to make friends. Some of those who relocate aim to obtain citizenship. Some people learn a language which becomes a secret way of communication.

By employing a combination of research strategies, we could explore in depth motivations and experiences of Russian speakers regarding language learning, thus contributing to a nuanced understanding of language learning processes within this linguistic community. For example, many immigrants recognize the practical necessity of learning the language of their new environment for everyday communication, accessing services, finding jobs, and integrating into society. Most participants see learning the local language as crucial for survival and success in their new home. They view it as a means of cultural adaptation and integration. They believe that acquiring proficiency in the local language allows them to better understand the culture and participate in events and rituals important for their new environment. These are signs that they accept societal norms of their new communities. They perceive language as a gateway to opportunities, such as higher education, career advancement, and upward social mobility. Some believe that children are considerably more successful in language learning than adults. Therefore, those who find it difficult to communicate in the local language in formal situation try to use their offspring as translators or interpreters, unaware that these language skills require special training and are, therefore, beyond the youngsters’ capabilities.

Immersion into the environment of their host countries made our participants value multilingualism and multiculturalism. While analysis of the collected texts testifies to L1 attrition, it is obvious that many Russophones prioritize maintaining proficiency in their native language as a way to preserve their cultural heritage and identity and are eager to transfer their L1 culture to the young generation (*cf.* Norton, 2000; Zabrodskaja and Ivanova, 2021). These efforts often meet staunch opposition of the youngsters, since members of the 1.5 and second generation immerse into the culture of the host country faster than their parents, and some are alienated from their family’s roots. This, as well as difficulties in the new language learning may cause adult immigrants’ frustration. Overwhelmed by the difficulty of acquiring proficiency in a new language and confronted with problems of social and occupational integration, adult immigrants tend to experience setbacks or feelings of inadequacy, which affects their attitude toward language learning and even re-evaluation of the decision to leave their home country. Understanding and addressing these issues is crucial for supporting immigrants in their language learning.

Policymakers should consult researchers studying local migrant communities and practitioners working with migrants in order to better understand specific cultural differences, fears and restrictions experienced by the adult immigrants in the process of language learning. They should develop and fund language programs, providing tailored support to enhance immigrants’ learning experience. They should acknowledge the importance of maintenance of immigrants’ L1s alongside the acquisition of the host country’s language, recognizing the cultural and cognitive benefits of multilingualism.

Educators have to design language courses that consider specific needs and backgrounds of adult learners, incorporating practical language use and cultural contexts to facilitate faster and more effective learning. Family-oriented language programs might address both parents and children, promoting a shared learning experience that can strengthen family bonds and improve outcomes for all generations (*cf.* Senyildiz, 2010). Community events and activities that encourage language practice in a supportive, real-world context help immigrants build confidence and social connections. Bridging the gap between immigrants’ native cultures and their new environments helps them discern cultural differences but also similarities, as a result reducing the emotional strain of integration.

## Data Availability

The data analyzed in this study is subject to the following licenses/restrictions: it is mostly publicly available. Portions of the data that do not compromise participant anonymity are available upon request from the author. Requests to access these datasets should be directed to ekaterina.protassova@helsinki.fi.
